# An Atypical Case of Varicella Meningitis in a Young Immunocompetent Male Patient With a Possible Concomitant Case of Ramsay-Hunt Syndrome

**DOI:** 10.7759/cureus.89299

**Published:** 2025-08-03

**Authors:** William B Harrington, Patrick Fugler, Vincent DeStefino

**Affiliations:** 1 Graduate Medical Education, University of Pittsburgh Medical Center, Altoona, USA; 2 Emergency Medicine, University of Massachusetts Chan Medical School (UMASS) Baystate Health, Springfield, USA

**Keywords:** benign aseptic meningitis, health care quality and cost, immunocompetent adult, ramsay-hunt syndrome, vzv meningitis

## Abstract

Varicella zoster virus (VZV) is a single-stranded enveloped RNA virus that is a common cause of chickenpox and herpes zoster. Herpes zoster (shingles) presents with a painful rash in a dermatomal distribution. Ramsay-Hunt syndrome (herpes zoster oticus) is a specific form of shingles, which occurs due to viral reactivation in the geniculate ganglion of cranial nerve VII. It can cause the triad of symptoms of ipsilateral facial paralysis, ear pain, and vesicles in the auditory canal or on the auricle. VZV is also a rare cause of aseptic meningitis, which occurs more commonly in immunodeficient rather than immunocompetent individuals. VZV meningitis can occur with or without the stereotypical rash, which may be very minimal if present. Here, we present a case of a 30-year-old immunocompetent man who presented to his local emergency department (ED) after failed treatment of otitis media and two syncopal events. ED evaluation was positive for nuchal rigidity, scalp tenderness, and an enlarged lymph node on his right posterior neck. PCR analysis was positive for VZV meningitis without overt rash. His only skin finding was erythematous patches in the right auditory canal with a bulging tympanic membrane. His auditory symptoms did not improve with adequate antibiotic treatment, meaning his symptoms were likely viral in nature. The treating infectious disease physician felt his auditory symptoms were related to his VZV infection; however, no confirmatory tests were completed. He was discharged on hospital day 4 and completed a total of 14 days of acyclovir 500mg three times a day. Additionally, we discuss the implications of intravenous acyclovir therapy in mild VZV meningitis in young immunocompetent individuals and the role oral valacyclovir therapy can play.

## Introduction

Varicella zoster virus (VZV) is a single-stranded enveloped RNA virus that is most synonymous with chickenpox and herpes zoster (shingles). While relatively uncommon, VZV can cause aseptic meningitis in immunocompetent individuals, accounting for 2.5-8% of aseptic meningitis cases [1]. When VZV meningitis occurs in an immunocompetent individual, they typically have multiple comorbidities or are older [2]. The severity of VZV meningitis can vary greatly and may present with or without concomitant herpes zoster infection [3]. For those individuals who have concomitant infections, the stereotypical rash associated with herpes zoster may be very discrete or even absent [2]. Regardless of whether the rash is present, if concomitant meningitis and shingles infection occur, patients will have pain in a dermatomal distribution [2].

Ramsay-Hunt syndrome (herpes zoster oticus) is a specific form of shingles that occurs from viral reactivation in the geniculate ganglion of cranial nerve VII. It commonly affects cranial nerves VII and VIII and can rarely affect cranial nerves V, IX, and X [2]. It can cause ipsilateral facial paralysis, ear pain, and vesicles in the auditory canal or on the auricle [2].

The current treatment recommendation from the Infectious Disease Society of America (IDSA) for VSV meningitis is intravenous (IV) acyclovir 500mg three times a day for 10-14 days [4]. However, as the severity of VZV meningitis can vary greatly, so can the treatment regimen. There are increasing case reports and meta-analyses to suggest non-severe VZV meningitis cases can be adequately treated with oral valacyclovir, if treatment is required at all [5,6]. Here we present a case of an immunocompetent 30-year-old man with acute VZV meningitis with concomitant otitis media, which was suspicious for an atypical presentation of Ramsay-Hunt syndrome. Additionally, we will discuss their anti-viral treatment and how this can impact social determinants of health.

## Case presentation

The patient is a 30-year-old married man from rural northwestern Pennsylvania. The patient is a construction salesman who additionally owns a dog kennel, breeds hunting dogs, and has hunting birds on his home property. He initially presented to a local urgent care with complaints of right ear pain and nasal congestion. He described his ear as sensitive to the touch and the pain as burning/throbbing/stabbing. Physical exam was positive for erythema of his right ear canal and ipsilateral bulging tympanic membrane. He was prescribed Augmentin 875/125mg twice daily for 10 days. After completing three days of antibiotics, he returned to the urgent care with a chief complaint of severe headache and no change in his ear pain. He described the headache as the worst of his life. It was an 8.5/10 on the pain scale, and he described it as a throbbing/stabbing pain. Additionally, he admitted to significant neck stiffness, scalp tenderness, and scalp pruritus. He had one near-syncopal event at home, and when the urgent care attempted to draw labs, he had a syncopal event. The urgent care physician felt his syncope was due to dehydration and referred the patient to the emergency department (ED) for IV hydration.

At his local ED, the patient’s chief complaint was “generally not feeling well.” His review of symptoms was positive for severe headache, ear pain, neck stiffness, scalp tenderness (right > left), and enlarged lymph node on his posterior neck. During the history of present illness, he admitted that he had recently been on a hunting trip where several people were diagnosed with atypical pneumonia after exposure to birds and ticks.

**Figure 1:**
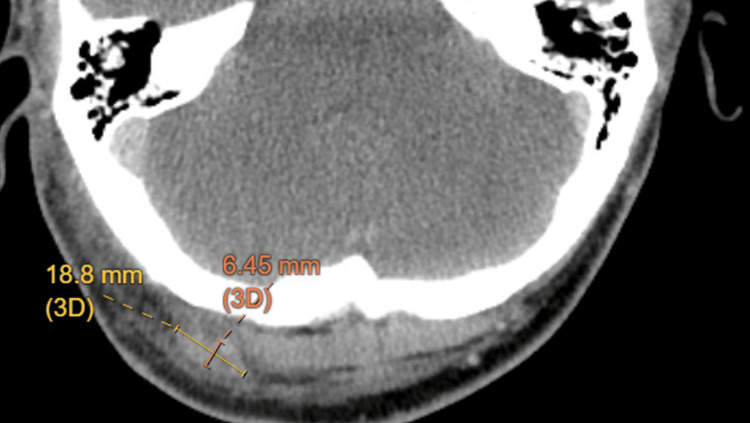
Axial image from CT soft tissue neck with intravenous contrast showing an enlarged right occipital lymph node measuring 18.8mm by 6.45mm.

**Figure 2:**
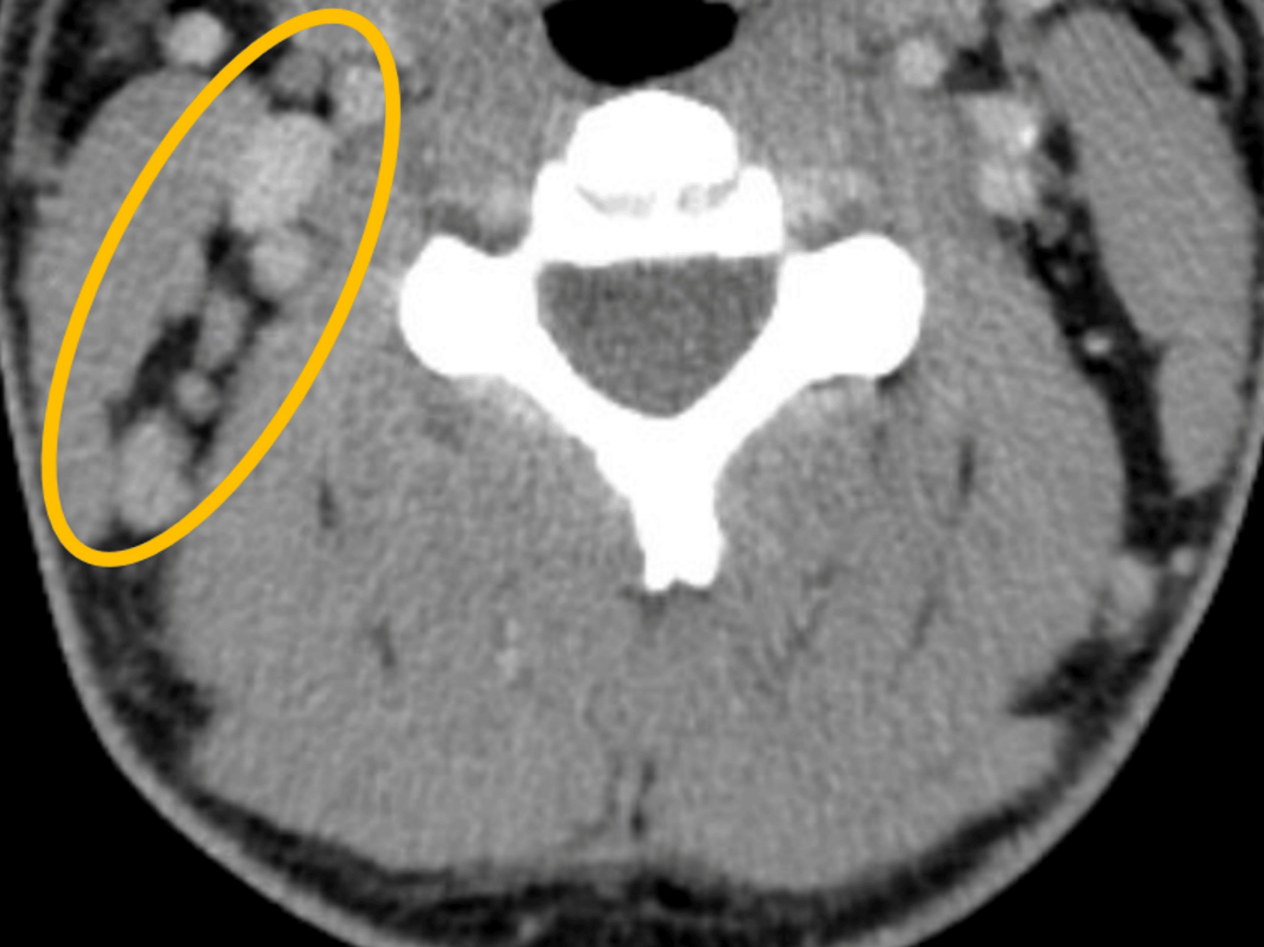
Axial image from CT soft tissue neck with intravenous contrast showing right-sided retro-auricular lymphadenopathy.

His physical exam was positive for nuchal rigidity, enlarged lymph node on the right side of his posterior neck, and bulging, erythematous right tympanic membrane with an erythematous auditory canal. The exam was negative for pain with horizontal head movement, ocular muscle movement, or photophobia. Vital signs were only significant for a temperature of 100.3°F. He was ordered stat a CT head without contrast and CT soft tissue neck with IV contrast in the ED, along with labs. CT non-contrast head was negative, but CT soft tissue confirmed a 6mm right occipital lymph node and right retro-auricular lymphadenopathy (Figures 1, 2). Labs included a complete blood count with differential, basic metabolic panel, lactate, and procalcitonin, and a lumbar puncture was performed for cerebrospinal fluid analysis. Initial results can be seen in Table 1. Lumbar puncture was atraumatic, but no opening pressure was obtained. 

**Table 1:** Significant lab results from CBC with differential, procalcitonin, and cerebrospinal fluid from the rural ED.

Lab	Value (normal range)	(Normal Range) Units
Procalcitonin	0.17	(0.0-0.10) ng/mL
Neutrophils	75.2	(42.0-75.0)%
Monocytes	11.4	(1.0-11.0) k/mm^3^
Cerebrospinal fluid		
Protein	58	(15-45) mg/dL
Glucose	59	(39-75) mg/dL
Lymphocytes	95%	(n/a)%
Total nucleated cells	16	(0-5) /mm^3^

In the ED, he was started on broad-spectrum coverage for bacterial meningitis with vancomycin 2g every 12 hours, ampicillin 2g once, and ceftriaxone 2g every 12 hours. The rural hospital did not have an infectious disease specialist; therefore, the patient was transferred to a regional health center.

Once he arrived at the regional hospital and had repeat labs, which were significant for increased neutrophils and C-reactive protein. Results of inpatient labs can be seen in Table 2. His preliminary CSF results were positive for VZV. At this time, the patient admitted to having chickenpox as a child and denied ever having episodes of shingles. He was started a 14-day course of IV acyclovir 500mg three times a day for VZV meningitis. Bacterial gram stain was negative; therefore, vancomycin was discontinued. His ceftriaxone was decreased to daily dosing, but not discontinued, as the Lyme testing panel had not resulted. Lyme disease was ruled out on hospital day 3, and ceftriaxone was subsequently discontinued.

**Table 2:** Abnormal labs from day hospital day 1 (HD1).

Lab	Value (normal range)	(Normal Range) Units
Neutrophils	87.80	(42.0-75.0)%
C-reactive protein	11	(0.0-5.0) mg/L

On his hospital day 2, his slightly abnormal labs normalized. The patient rapidly improved, and his neck pain was completely resolved by hospital day 3. However, he continued to have right ear pain, scalp tenderness, and scalp pruritus. Repeat examination of the ear again showed an erythematous, bulging right tympanic membrane. Additionally, there were patches of erythema in the auditory canal, but no discrete lesion was appreciated. The infectious disease physician felt this was associated with his VZV infection. However, no confirmatory testing was completed.

**Figure 3:**
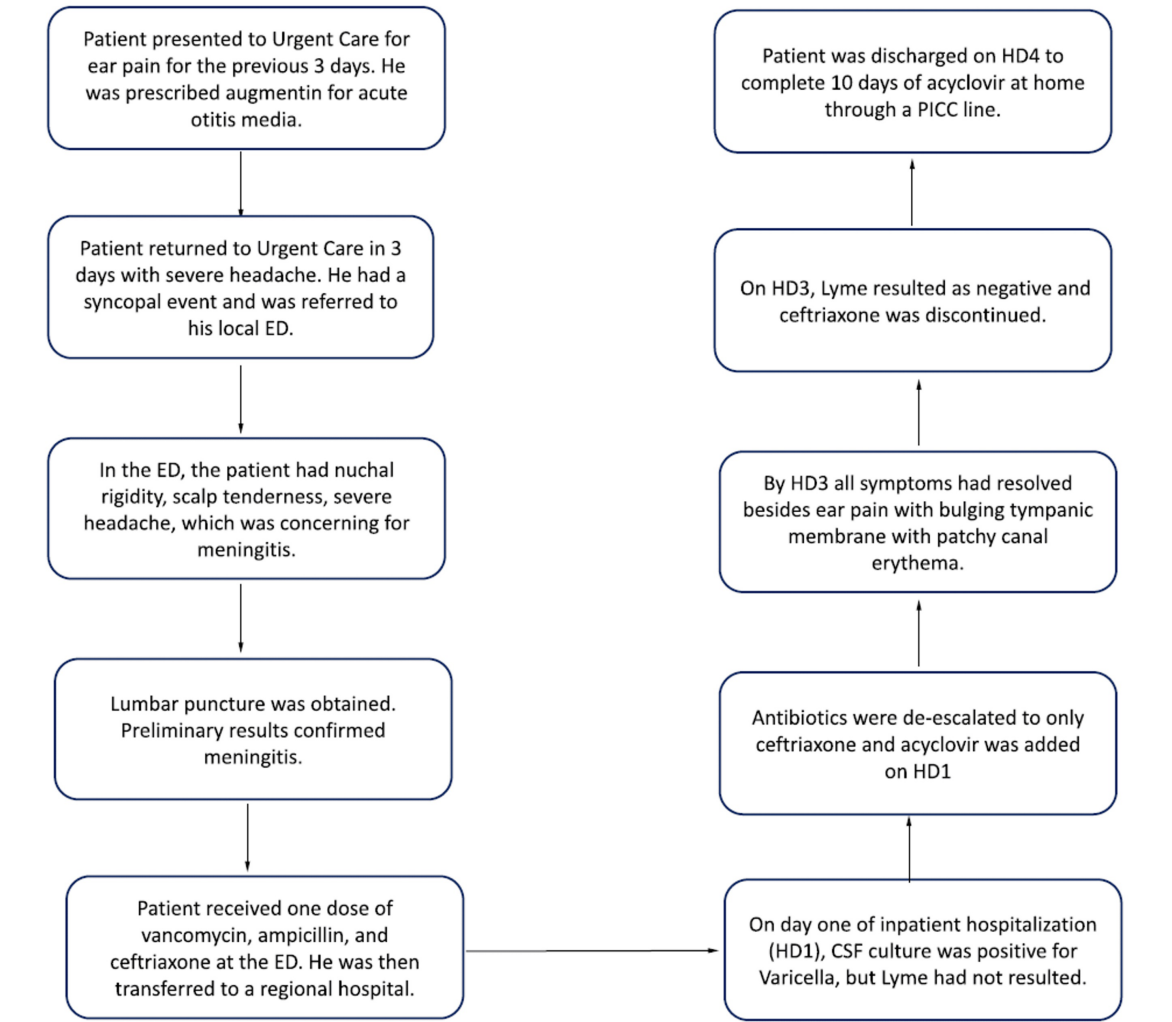
A flow chart of events related to the patient's illness onset and hospitalization.

His ear pain was treated symptomatically with acetaminophen and flonase, which provided symptomatic relief. He was discharged on hospital day 4 and was sent home with home nursing to complete the remaining 10 days of IV acyclovir. He completed his remaining therapy without complication, and he fully recovered without sequela. A flow chart of events can be found in Figure 3. 

## Discussion

Central nervous system (CNS) infection with VZV affects predominantly elderly and immunocompromised patients. CNS involvement in young healthy immunocompetent adults is rare, with only a few cases described. Additionally, while a majority of VZV meningitis cases present with typical concomitant pruritic vesicular rash, the incidence is not well-defined in the literature. Here we report a case of a young immunocompetent patient who presented with right-sided ear pain followed by scalp tenderness, scalp pruritus, severe headache, and nuchal rigidity without the characteristic rash. This emphasizes the need for high clinical suspicion and appropriate diagnostic testing even in healthy individuals with the absence of typical presenting symptoms.

Traditionally, VZV meningitis has been treated with IV acyclovir for 14 days for immunocompetent individuals, and this was the antiviral regimen prescribed to this patient [6]. However, there is literature to suggest that patients who have a minor case of VZV meningitis, are not willing/unable to complete IV antivirals, or are not candidates for PICC lines can be treated with oral valacyclovir [5]. Pharmacological data suggest that oral valacyclovir 500mg every six hours has similar or better CNS bioavailability to IV acyclovir 500mg three times a day [7-11]. As this patient had minor symptoms with normal infectious markers and his symptoms resolved by hospital day 3, he would have likely been successfully treated with oral valacyclovir. If he were treated orally, he would have had significantly less healthcare-associated costs from his illness.

There are several reasons why this patient would have benefited from oral therapy. One of which was the mild severity of the patient’s condition. During his four-day hospitalization, the patient was only febrile once, never had true leukocytosis, and had negative lactate and procalcitonin levels. Additionally, the average admission length for VZV meningitis in immunocompetent individuals is seven days, but this patient was discharged in four days [6]. Literature suggests that for immunocompetent individuals with mild VZV meningitis, there was no difference in disease outcomes for those treated with antivirals vs those who did not receive any treatment other than symptom management [5].

As the patient was receiving IV antivirals, he was discharged with a PICC line. There is a risk of PICC line infections in the hospital when trained professionals are caring for them [12]. This risk only increases when the patient is caring for their own central line. Besides the risk of infection, there is an increased risk for venous thrombosis or occlusion of the PICC line [13]. This patient did experience PICC line occlusion, resulting in a second PICC line being placed. Additionally, this patient lived on a small farm where they would breed rabbits and hunting dogs. Due to their breeding of hunting dogs, they had various bird species for training purposes. Recreationally, the patient rode horses, and he was responsible for cleaning their stalls. He also had four indoor cats and seven dogs that lived in his house. All this being said, he had an increased risk of PICC line infection even if receiving the best education on infection prevention. With oral treatment, this risk would be mitigated.

There is a significant healthcare cost associated with patients discharged with a PICC line. This patient was required to stay an extra day in a specialty unit simply to arrange home nursing for his IV management. Once the patient was discharged home, he had home nursing come once a day for the first three days, followed by every third day until he completed his antiviral regimen. In total, the patient received a bill for his inpatient admission and subsequent antiviral treatment, which totaled close to $8,000 (roughly $2,000 was the cost of his outpatient IV treatment). Assuming each day of his four-day admission was equal, he could have saved ~$4,000 ($2,000 from hospital stay and $2,000 from home nursing) from his bill, if treated orally. As previously mentioned, there have been case reports of patients who have been successfully treated with oral valacyclovir after a minimum of two-day hospitalization [5]. This patient did warrant inpatient admission to diagnose his meningitis but likely could have been discharged home once bacterial meningitis was ruled out (hospital day 3).

While not related to antiviral management, another area of discussion for this case is the possibility of the patient concomitantly having an atypical presentation of Ramsay-Hunt syndrome. Ramsay-Hunt syndrome is the activation of shingles, typically in cranial nerves VII and VIII [14]. In most cases, this is extremely painful and presents with a stereotypical rash. However, unlike normal VSV infections, the rash in VZV meningitis can be very discrete or not visualized [2]. Initially, this patient presented to the urgent care with a focal complaint of ear pain. This patient received Augmentin for three days, vancomycin for one day, one dose of ampicillin, and ceftriaxone for three days (a total of six days of antibiotics) without any improvement in his auditory symptoms. As the most common causes of otitis media are *Streptococcus Pneumoniae *and* Haemophilus Influenzae, *this patient would have been adequately treated with those antibiotics [15]. As his auditory symptoms did not improve with an appropriate antibiotic regimen, this would suggest his otitis media was viral in nature. While there was no confirmatory testing or biopsy, it seems highly probable that his otitis media was secondary to VZV infection. As mentioned, the treating infectious disease physician felt his auditory symptoms were related to his VZV infection. His otitis media was symptomatically treated with steroids and acetaminophen and completely resolved by the end of his antiviral treatment.

## Conclusions

In conclusion, we presented a case of VZV meningitis with possible concomitant Ramsay-Hunt syndrome in a young, immunocompetent male patient with no substantial co-morbidities. Additionally, we add support that mild cases of VZV meningitis can be treated with oral valacyclovir and therefore could substantially decrease healthcare-related costs. This should be evaluated on a case-by-case basis and could be addressed at a hospital follow-up appointment. Lastly, this case emphasizes the need for high clinical suspicion and appropriate diagnostic testing, even if the patient presents with atypical symptoms. 
